# Clinical hypothermia temperatures increase complement activation and cell destruction via the classical pathway

**DOI:** 10.1186/1479-5876-12-181

**Published:** 2014-06-24

**Authors:** Tushar A Shah, Clifford T Mauriello, Pamela S Hair, Amandeep Sandhu, Michael P Stolz, William Thomas Bass, Neel K Krishna, Kenji M Cunnion

**Affiliations:** 1 Department of Pediatrics, Eastern Virginia Medical School, 855 West Brambleton Avenue, Norfolk, VA 23510, USA; 2 Department of Microbiology and Molecular Cell Biology, Eastern Virginia Medical School, 700 West Olney Road, Norfolk, VA 23507-1696, USA; 3 Children’s Specialty Group, 811 Redgate Avenue, Norfolk, VA 23507, USA; 4 Children’s Hospital of The King’s Daughters, 601 Children’s Lane, Norfolk, VA 23507, USA

**Keywords:** Therapeutic hypothermia, Complement, Classical pathway, Complement inhibitor, Ischemia-reperfusion injury, Inflammation

## Abstract

**Background:**

Therapeutic hypothermia is a treatment modality that is increasingly used to improve clinical neurological outcomes for ischemia-reperfusion injury-mediated diseases. Antibody-initiated classical complement pathway activation has been shown to contribute to ischemia-reperfusion injury in multiple disease processes. However, how therapeutic hypothermia affects complement activation is unknown. Our goal was to measure the independent effect of temperature on complement activation, and more specifically, examine the relationship between clinical hypothermia temperatures (31–33°C), and complement activation.

**Methods:**

Antibody-sensitized erythrocytes were used to assay complement activation at temperatures ranging from 0-41°C. Individual complement pathway components were assayed by ELISA, Western blot, and quantitative dot blot. Peptide Inhibitor of complement C1 (PIC1) was used to specifically inhibit activation of C1.

**Results:**

Antibody-initiated complement activation resulting in eukaryotic cell lysis was increased by 2-fold at 31°C compared with 37°C. Antibody-initiated complement activation in human serum increased as temperature decreased from 37°C until dramatically decreasing at 13°C. Quantitation of individual complement components showed significantly increased activation of C4, C3, and C5 at clinical hypothermia temperatures. In contrast, C1s activation by heat-aggregated IgG decreased at therapeutic hypothermia temperatures consistent with decreased enzymatic activity at lower temperatures. However, C1q binding to antibody-coated erythrocytes increased at lower temperatures, suggesting that increased classical complement pathway activation is mediated by increased C1 binding at therapeutic hypothermia temperatures. PIC1 inhibited hypothermia-enhanced complement-mediated cell lysis at 31°C by up to 60% (P = 0.001) in a dose dependent manner.

**Conclusions:**

In summary, therapeutic hypothermia temperatures increased antibody-initiated complement activation and eukaryotic cell destruction suggesting that the benefits of therapeutic hypothermia may be mediated via other mechanisms. Antibody-initiated complement activation has been shown to contribute to ischemia-reperfusion injury in several animal models, suggesting that for diseases with this mechanism hypothermia-enhanced complement activation may partially attenuate the benefits of therapeutic hypothermia.

## Introduction

Therapeutic hypothermia improves neurological outcome in ischemia-reperfusion injury (IRI) caused by cardiac arrest [1], traumatic brain injury [2], stroke [3], acute liver injury [4] and neonatal hypoxic ischemic encephalopathy [5]. In addition to decreased cerebral metabolism [6], hypothermia appears to attenuate multiple processes that contribute to tissue damage following IRI including neuroinflammation [7], excitotoxicty [8], mitochondrial dysfunction [9], apoptosis [10], free radical production [11], seizure activity [12] and blood-brain barrier disruption [13]. Hypothermia is also purported to stimulate protective cold shock proteins [14].

The complement system is an extremely potent inflammatory cascade composed of more than 30 plasma and cell membrane proteins that plays major roles in innate immune defense as well as many inflammatory diseases including IRI. Clinical and experimental studies in multiple organ systems have shown that reperfusion following ischemia results in local activation of the complement system [15]. Complement activation in IRI is primarily mediated by circulating natural antibodies (IgM) binding to neo-antigens expressed on the surface of hypoxia-stressed endothelial cells leading to classical complement pathway activation or lectin pathway activation [16,17]. In addition to direct complement-mediated endothelial cell damage during reperfusion, complement induced neutrophil recruitment and activation causes significant injury through the phagocytic actions of macrophages, free radical production and synthesis of toxic products [8]. Complement inhibition has been shown to be protective in animal models of brain [18], kidney [19] and liver [20] IRI.

While several mechanisms of action for therapeutic hypothermia have been proposed, the effect of hypothermia on complement activation is unknown. Whether improved outcomes in IRI could be due, in part, to hypothermia inhibiting or attenuating complement activation has not yet been explored. Such a mechanism is plausible given that complement activation is enzymatic and slows down dramatically at very low temperatures of 0 – 4°C [21,22]. However, whether the temperatures of clinical hypothermia, 31 - 33°C, would inhibit complement activation remains untested. The increasing use of therapeutic hypothermia in the treatment of IRI diseases makes understanding the contribution of the complement system critical. These experiments elucidate how therapeutic hypothermia temperatures modulate antibody-initiated complement activation.

## Methods

### Ethics statement

Pooled normal human serum (NHS) was derived from the blood of healthy human volunteers obtained with written informed consent in accordance with an Institutional Review Board approved protocol (IRB 02-06-EX-0216, Eastern Virginia Medical School).

### Materials

Sheep erythrocytes were purchased from MP Biomedicals, LLC. Anti-sheep-erythrocyte-IgM was purchased from Rockland Incorporated. Sheep erythrocytes were incubated with antibody at 30°C to generate antibody-sensitized cells (EA), as previously described [23]. All antibody-sensitization was performed at 30°C. Standard complement buffers were prepared for hemolytic assays: GVBS^++^ (Veronal buffered saline, 0.1% gelatin, 0.15 mM CaCl_2_, and 1.0 mM MgCl_2_), Mg-EGTA-GVBS (Veronal buffered saline with 5 mM MgCl_2_ and 8 mM EGTA), and GVBS^- -^ (Veronal buffered saline, 0.1% gelatin, 0.01 M EDTA), Veronal Buffered Saline (10 mM Barbital, 145 mM NaCl, pH 7.4). Peptide Inhibitor of Complement C1 (PIC1) version AcPA, described elsewhere [24], was synthesized by New England Peptide (Gardner, MA). Lyophilized PIC1 was reconstituted in DMSO. Pooled normal human serum (NHS) was prepared as previously described [25]. C8-depleted human serum and purified C1q were purchased from CompTech (Tyler, TX).

### Hemolytic assays

Antibody-initiated complement hemolytic assays were performed by incubating 100 μL EA with 0.2% NHS in GVBS^++^. Hemolytic assays were performed for one hour at temperatures ranging from 0°C to 37°C. A separate assay was performed with more clinically relevant temperatures of 31°, 33°, 35°, 37°, 39°, and 41°C. EA were also incubated with water or buffer as controls. Hemolysis reactions were quenched with 4 mL GVBS^- -^ and the supernatants recovered after sedimentation. Hemolysis was quantified by spectrophotometry of the supernatants at 412 nm and normalized as previously described [23]. Alternative complement pathway hemolytic assays were performed by incubating rabbit erythrocytes (CompTech, Tyler, TX) with 4% NHS in Mg-EGTA-GVBS at temperatures ranging from 0°C to 37°C. A separate assay was performed with more clinically relevant temperatures of 31°, 33°, 35°, 37°, 39°, and 41°C. Reactions were quenched with 2 mL GVBS^- -^, sedimented and measured as above.

### C5a ELISA

Samples were generated by incubating 1% NHS with 400 μg/ml heat-aggregated IgG in GVBS^++^ at each temperature for one hour. Heat-aggregated IgG was generated from purified human IgG (Gammagard, Baxter) as described elsewhere [23]. The reaction was stopped with EDTA-GVBS^- -^ and the samples were measured by C5a ELISA (R&D Systems). Background values from controls of NHS and heat-aggregated human IgG incubated on ice for one hour were subtracted.

### C3 and C4 assays

C3/C4 samples were generated by incubating C8-deficient serum (to prevent hemolysis), 1% final, with 0.25 mL EA in a total of 0.75 mL of GVBS^++^ for one hour at each temperature. After incubation, the samples were washed twice with water to remove hemoglobin, and the resulting membrane bound C3/C4 was stripped using 25 mM methylamine. These samples were quantitated by total C3 ELISA, iC3b ELISA, and C4 ELISA as previously described [26]. C3 and C4 Western blot analysis was performed as described elsewhere [26].

### C1s western blot

A Western blot to assess C1s activation by heat aggregated IgG has been previously described [27,28] One microliter of partially purified C1 (0.2 mg/ml, Complement Technologies, Inc.), 5 μl of heat-aggregated IgG (1:250 dilution from 50 μg/ml stock), and 5 μl of PBS were combined on ice and samples incubated at the following temperatures: 31, 33, 35, 37, 39 and 41°C for 0, 30, 45, 60 and 90 minutes. After incubation, 4 μl of loading buffer was added and each sample was boiled. Gel electrophoresis on a 10% SDS-PAGE gel was performed at 140 V for 60 minutes. The gel was then transferred to a nitrocellulose membrane at 200 mA for 60 minutes. The membrane was washed twice for five minutes in PBS buffer and then blocked overnight in 5% NFDM-PBS. The next day, the membrane was probed with primary antibody, Goat anti-C1s (Quidel), at a dilution of 1:2000 in 5% NFDM-PBS/0.1% Tween-20 for one hour at room temperature. The membrane was washed 3X for 10 minutes with PBS/0.1% Tween-20. Then, the membrane was probed with secondary antibody, Donkey anti-goat IR680 (Li-cor Biosciences), at a dilution of 1:10,000 in 5% NFDM-PBS/0.1% Tween-20 for one hour at room temperature. After washing the membrane 3X with PBS/0.1% Tween-20, it was imaged on an Odyssey imager to determine the amounts of the C1s heavy and light chains characteristic of activated C1s relative to the proenzyme species.

### C1/C1q binding

C1 either from NHS (0.33% final) or C8-deficient serum (CompTech), or purified C1q (Comp Tech) at 0.24 μg/ml final, was combined with 100 μl of sensitized sheep erythrocytes in a total volume of 0.75 ml of GVBS++ for one hour at the various temperatures. These cells were then washed twice with water and the resulting cell pellet was dissolved with 0.5% NP40. Membrane bound C1q was analyzed by a quantitative Dot Blot assay. Pure C1q was titrated onto PVDF membrane, along with the samples, via a Dot Blot apparatus. The membrane was blocked with 3% BSA, probed with a goat anti-C1q antibody (Comp Tech), and then an HRP-labeled rabbit anti-goat secondary antibody (Sigma-Aldrich). Bound antibody was detected by enhanced chemiluminescence. Using the Quantity One software (Bio-Rad), grey scale values were assigned to the pure C1q titration and the samples so that a linear regression could be used to quantify the amount of C1q in each sample.

### PIC1 inhibition of cold-enhanced complement activation

Peptide Inhibitors of complement C1 (PIC1) specifically inhibit C1 activation, as previously described [24,28]. Here we use the AcPA form (IALILEPICCQERAA) [24] of PIC1. Preparations of 16.5% normal human serum (NHS) in GVBS^++^ were incubated with 0.98 mM, 0.82 mM, 0.65 mM, 0.48 mM or 0.32 mM PIC1, DMSO vehicle control, or buffer control at 4°C for 1 hour. Samples were further diluted 1:10 in GVBS^++^. Hemolysis reactions were then performed with 100 μL of NHS sample plus 550 μL of additional GVBS^++^ and 100 μL of EA over 1 hour at either in 31° or 37°C. Controls and samples were processed and measured by spectrophotometry at 412 nm.

### Statistical analysis

Means and standard error of the means (SEM) were calculated from independent experiments. Statistical comparisons were made using one way ANOVA and Student’s *t*-test where appropriate. Statistical analysis was performed using GraphPad InStat 3 software.

## Results

### Functional hemolytic assays of complement activation at hypothermic temperatures

**Figure 1:**
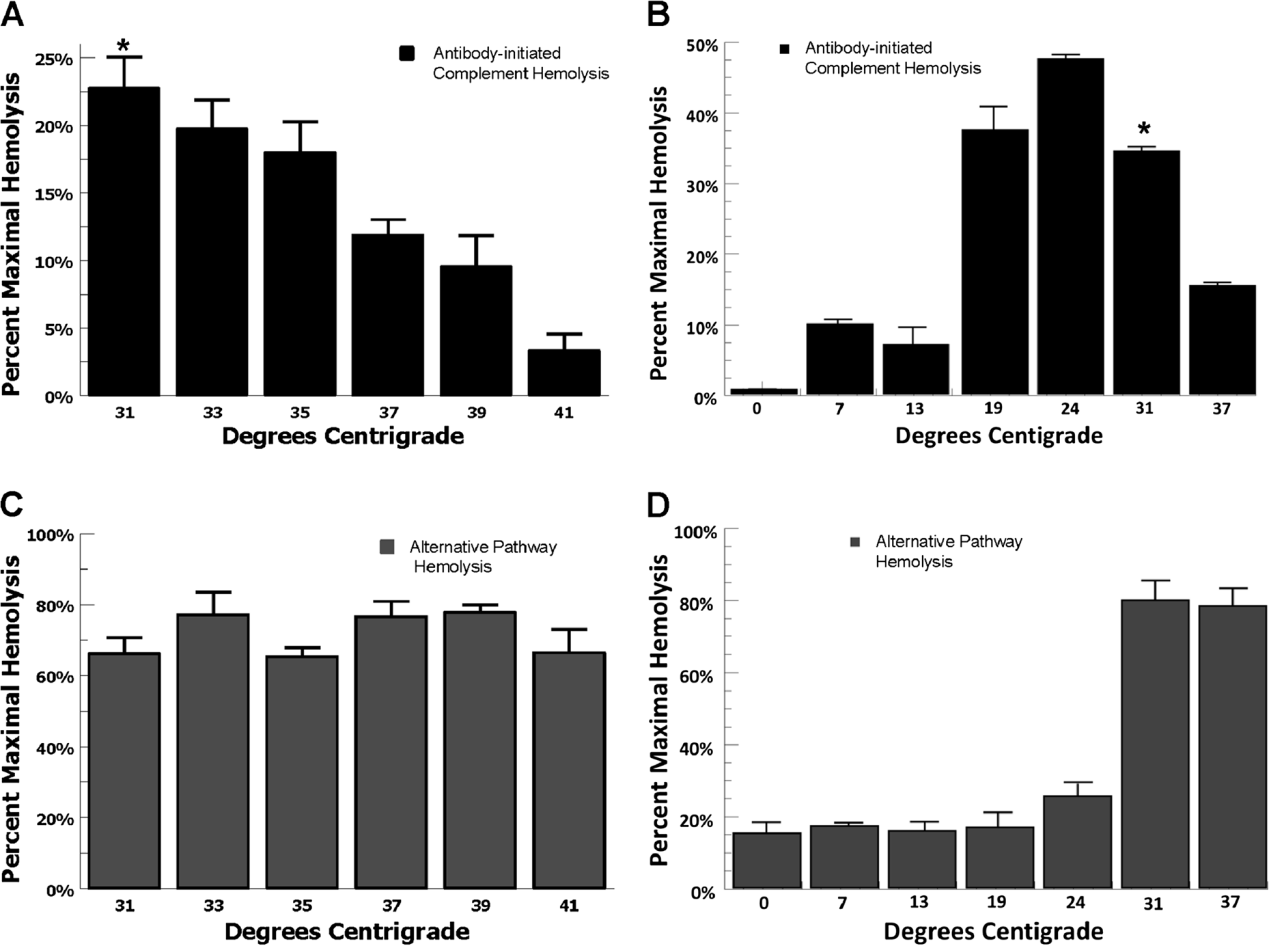
**Functional hemolytic assays of complement activation. (A)** CH50-type antibody-initiated complement assays incubating antibody-sensitized erythrocytes (EA) with 0.2% human serum for 1 hour at 31-41°C showed that hemolysis of antibody-sensitized erythrocytes in human serum differed significantly as a function of temperature (ANOVA P < 0.01). There is 2-fold more hemolysis at 31°C vs. 37°C (*t-test P = 0.01) (P ≤ 0.05 for the comparisons 31 vs. 37, 31 vs. 39, 31 vs. 41, 33 vs. 37, 33 vs. 39, 33 vs. 41, 35 vs. 39, 35 vs. 41, with significantly more hemolysis at lower temperatures) Data are mean ± SEM for 4 independent experiments. **(B)** Evaluating antibody-initiated complement activation from 0-37°C showed that hemolysis of antibody-sensitized erythrocytes in human serum differed significantly as a function of temperature (ANOVA P < 0.01). There was 2.5-fold more hemolysis at 31°C vs. 37°C (*t-test P = 0.01). (P ≤ 0.05 for the comparisons 31 vs. 37, 24 vs. 37, 19 vs. 37, with significantly more hemolysis at lower temperatures). The trend of increased complement activation was reversed at 13°C. (No statistical difference between hemolysis at 13 vs. 37 or 7 vs. 37). Data are mean ± SEM for 4 independent experiments. **(C)** AP50-type alternative complement pathway assays, incubating rabbit erythrocytes with 4% human serum in Mg-EGTA-GVBS for 30 minutes at 31-41°C showed that temperature did not influence complement-mediated cell lysis over therapeutic hypothermia temperatures (ANOVA P = 0.45). Data are mean ± SEM for 4 independent experiments. **(D)** Evaluating alternative pathway activation from 0-37°C showed that alternative pathway activation was significantly inhibited at 24°C and below. There was no difference in the degree of hemolysis at 31°C vs. 37°C) Data are mean ± SEM for 4 independent experiments.

In order to evaluate antibody-initiated complement-activation at therapeutic hypothermia temperatures, we tested antibody-sensitized sheep erythrocytes (EA) incubated in normal human serum at 31°C-41°C and measured hemolysis. In this CH50-type assay, hemolysis of antibody-sensitized erythrocytes in human serum differed significantly as a function of temperature (ANOVA P < 0.01). There was 9-fold more hemolysis at 31°C compared with 41°C (*t*-test P < 0.01) and 2 fold more hemolysis at 31°C compared with 37°C (*t*-test P = 0.01) (Figure 1A). These results show that antibody-initiated complement activation increased at therapeutic hypothermia temperatures. Given that complement activation is severely inhibited at near 0°C temperatures, we then evaluated complement activation for temperatures ranging from 0°C-37°C. Antibody-initiated complement activation in human serum increased as temperature decreased from 37°C to 24°C until dramatically decreasing at 13°C (Figure 1B). In these assays a similar 2-fold increase in antibody-initiated complement activation was noted comparing 31°C with 37°C (*t*-test P < 0.01). From 0°C – 13°C complement activation was minimal.We then tested whether similar effects would be observed for the alternative complement pathway over therapeutic hypothermia temperatures. In an AP50-type alternative complement pathway assay, temperature did not alter complement-mediated cell lysis at therapeutic hypothermia temperatures (ANOVA P = 0.45) (Figure 1C). Testing alternative pathway activation from 0°C – 37°C demonstrated that activation was significantly inhibited at 24°C and below (Figure 1D). These results show that antibody-initiated complement activation increased at clinical hypothermia temperatures compared with euthermia, suggesting that antibody-initiated complement activation occurring during reperfusion at hypothermic temperatures could potentially increase complement-mediated tissue damage.

### Complement cascade activation assays

**Figure 2:**
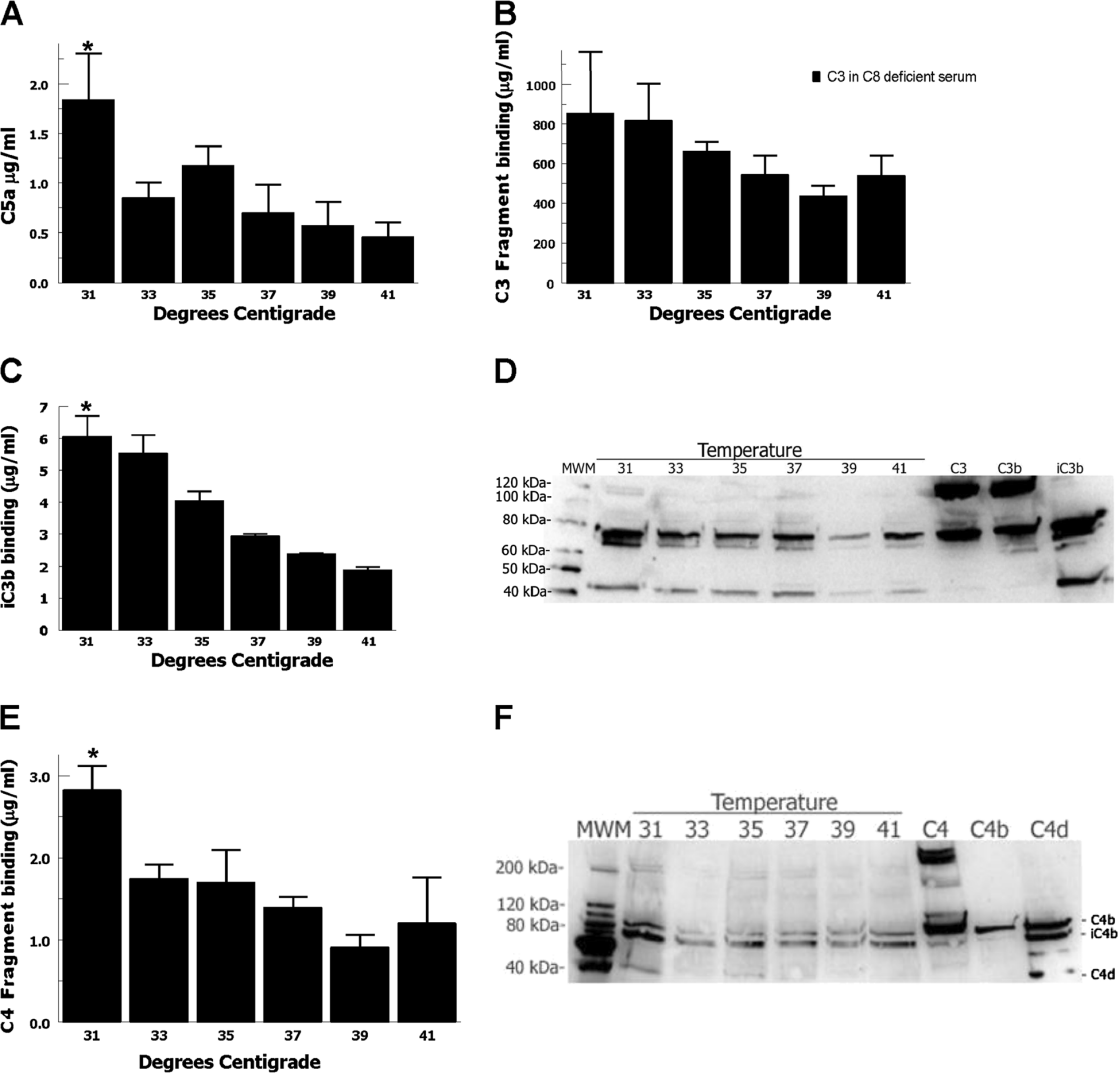
**Complement cascade activation assays. (A)** Heat-aggregated IgG 1% normal human serum for 1 hour at 31-41°C demonstrated that C5a generation differed significantly as a function of temperature (ANOVA P < 0.01). There was a 2.5 fold increase in C5a generation at 31°C vs. 37°C (*t-test P =0.05). Data are mean ± SEM for 4 independent experiments. C3/C4-analysis samples were generated by incubating 1% C8-deficient serum (to prevent hemolysis) with 0.25 mL EA for one hour at 31-41°C. **(B)** Total C3-fragment opsonization did not differ significantly as a function of temperature (ANOVA P = 0.5). **(C)** Cell membrane bound iC3b differed significantly as a function of temperature (ANOVA P < 0.01), with a 2-fold increase at 31°C vs. 37°C (*t*-test P = 0.04). Data are mean ± SEM for 4 independent experiments. **(D)** A representative Western blot analysis of membrane-bound C3-fragments confirmed that iC3b was the predominant form. **(E)** C4-fragment opsonization differed significantly as a function of temperature (ANOVA P = 0.04). There was an almost 2-fold increase in C4 generation at 31°C vs. 37°C (*t-test P = 0.05). Data are mean ± SEM for 4 independent experiments. **(F)** A representative Western blot analysis demonstrated a mixture of C4b, iC4b and C4d fragments.

Complement-mediated hemolysis results from pores generated by the membrane attack complex (MAC), suggesting that increased terminal complement cascade activation was occurring at lower temperatures. In order to further evaluate activation of the terminal complement cascade we measured C5a generation in response to a different antibody-initiated complement activator, heat-aggregated IgG. C5a is also an extremely potent anaphylatoxin important for phagocytic cell recruitment and activation [29] and implicated in contributing to tissue damage in many inflammatory diseases processes [30]. Heat-aggregated IgG incubated with normal human serum demonstrated that C5a generation differed significantly as a function of temperature (ANOVA P < 0.01). There was a >2-fold increase in C5a generation at 31°C compared with 37°C (*t*-test P =0.05) (Figure 2A). These data show that at clinical hypothermia temperatures terminal complement cascade activation increased, consistent with the CH50-type assay, and suggests that increased C5a generation at these temperatures could increase neutrophil recruitment and activation enhancing inflammation and host tissue damage during reperfusion.

C3 is the central component of the complement cascades and its activation results in generation of the anaphylatoxin C3a and opsonization of targets with the C3-fragments, C3b and iC3b. We tested C3 activation and opsonization with C3b/iC3b by incubating EA with C8-deficient serum (to prevent hemolysis). Though total C3-fragment opsonization as a function of temperature was not statistically significant (ANOVA P = 0.5) (Figure 2B), cell membrane bound iC3b differed significantly as a function of temperature (ANOVA P < 0.01), with a 2-fold increase at 31°C versus 37°C (*t*-test P = 0.04) (Figure 2C). Western blot analysis of membrane-bound C3-fragments confirmed that iC3b was the predominant form and suggested increased iC3b deposition at lower temperatures (Figure 2D) consistent with ELISA data. These data show that C3 activation and iC3b opsonization increased at clinical hypothermia temperatures, suggesting that these temperatures could increase opsonization of host cells (e.g. endothelial cells) during reperfusion targeting them for phagocyte attack.

In order to specifically evaluate the classical complement pathway, we tested C4 activation by incubating EA with C8-deficient serum. C4-fragment binding differed significantly as a function of temperature (ANOVA P = 0.04). There was an almost 2-fold increase in C4 generation at 31°C compared with 37°C (*t*-test P = 0.05) (Figure 2E). Western blot analysis demonstrated a mixture of C4b, iC4b and C4d fragments (Figure 2F) and suggested increased C4-fragment binding at 31°C. These findings show that classical pathway activation is increased at therapeutic hypothermia temperatures and suggests that C1 activation, which cleaves C4, is also increased at these temperatures.

### C1 activation and binding

**Figure 3:**
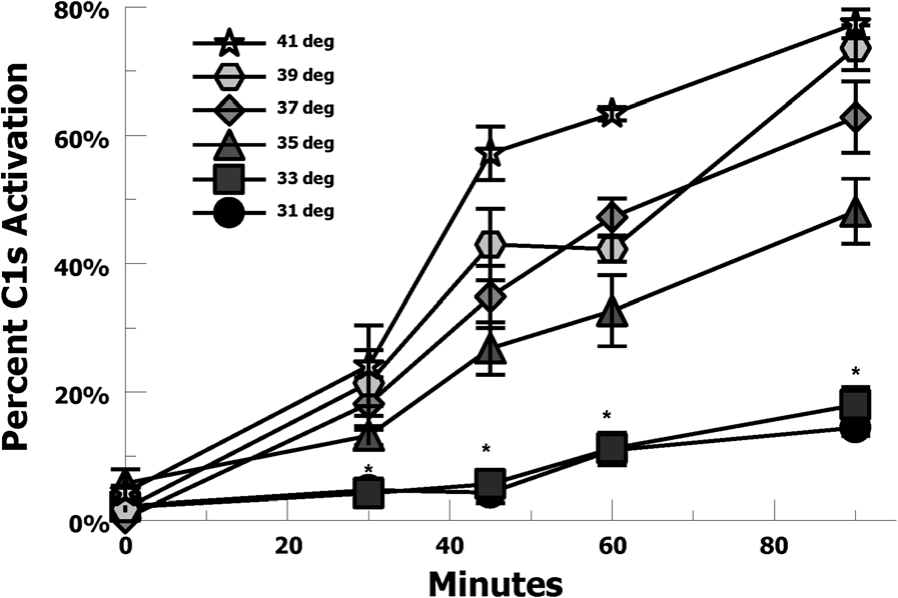
**C1s activation by heat-aggregated IgG for increasing temperatures.** One μg of partially purified human C1 was incubated with heat-aggregated IgG at 31-41 C for 0–90 minutes. C1s activation, as measured by generation of C1s heavy and light chains by Western blot methodology, was quantified by Odyssey imaging and demonstrated decreased activation of C1s at therapeutic hypothermia temperatures (ANOVA P < 0.01), with a 5-fold decrease at 31°C vs. 37°C (*t-test P < 0.01). Data are mean ± SEM for 3 independent experiments.

**Figure 4:**
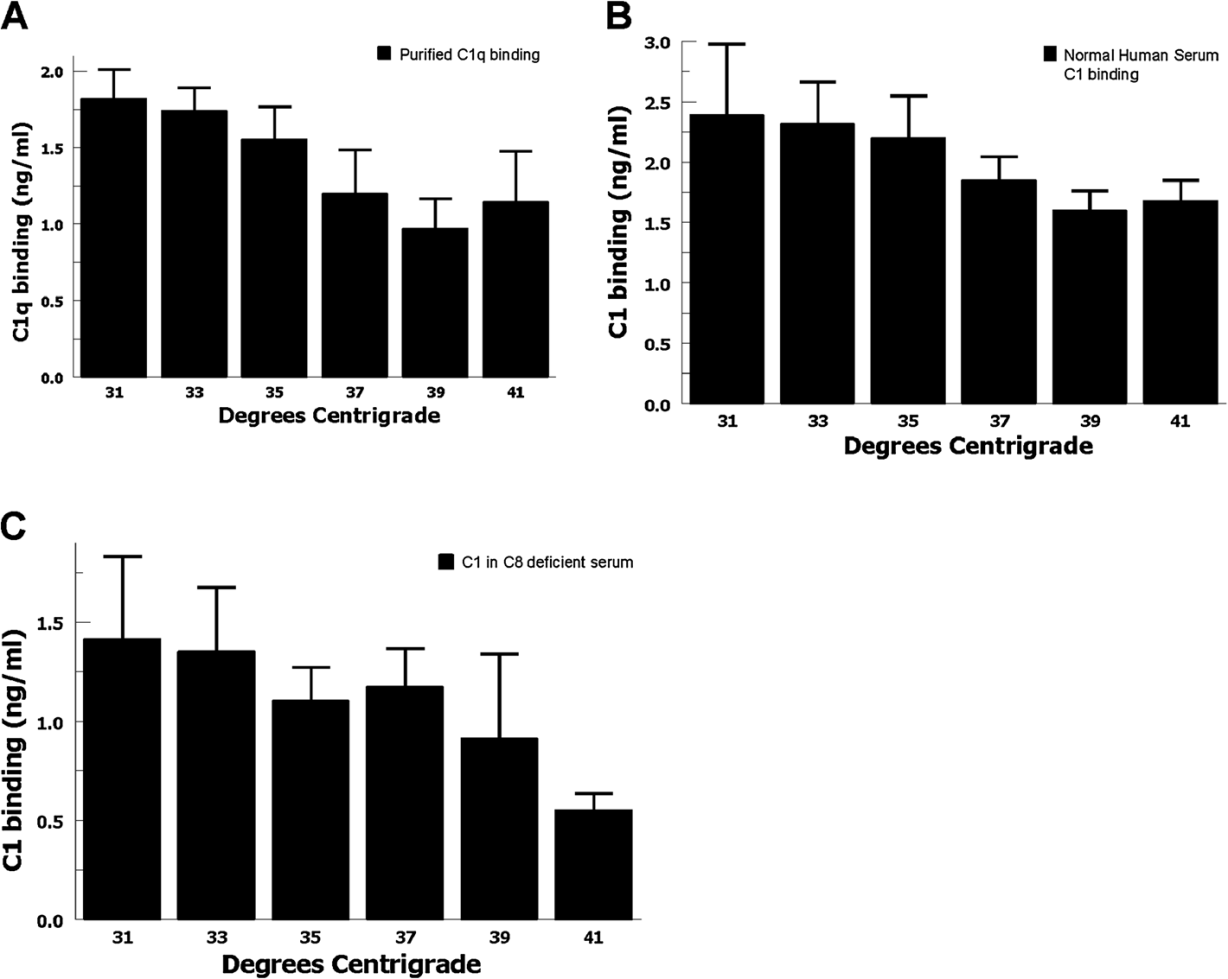
**C1/C1q binding at therapeutic hypothermia temperatures.** C1q binding was tested by incubating antibody-sensitized erythrocytes with **(A)** purified C1q, **(B)** normal human serum, or **(C)** C8-deficient serum for one hour at 31-41°C. In each case, there was a consistent trend of increased C1q binding to EA at lower temperatures. Data are mean ± SEM for 4 independent experiments.

C1 is a complex molecule consisting of a pattern (e.g., IgG/M) recognition component C1q, which associates with the serine protease tetramer C1r-C1s-C1s-C1r. When the C1q portion of the molecule binds to IgG/M, it causes auto-activation of the enzymatic tetramer leading to activation of C4 and the classical pathway cascade. Therefore, increased activation of C1 at lower temperatures could be caused by increased C1q binding, or increased enzymatic activity, or both. C1 enzymatic activation was tested at increasing temperatures over a 90 minute period using heat-aggregated IgG incubated with partially purified C1. C1s activation was assayed by the conversion of precursor C1s to its activated heavy and light chain forms, as previously described [27,28]. C1s activation decreased at therapeutic hypothermia temperatures, suggesting decreased enzymatic activity at lower temperatures (Figure 3).C1q and C1 binding was tested by incubating EA with purified C1q, or normal human serum, or C8-deficient serum. In each case, C1/C1q binding to EA showed a consistent trend of increasing at lower temperatures (Figures 4A, B, and C), suggesting that increased classical complement pathway activation is likely mediated by increased C1 binding at therapeutic hypothermia temperatures. These findings may also explain the lack of hypothermia-enhanced alternative pathway activation where initiation is not mediated by a pattern recognition molecule (e.g. C1q) binding event.

### Inhibition of C1-mediated complement activation

**Figure 5:**
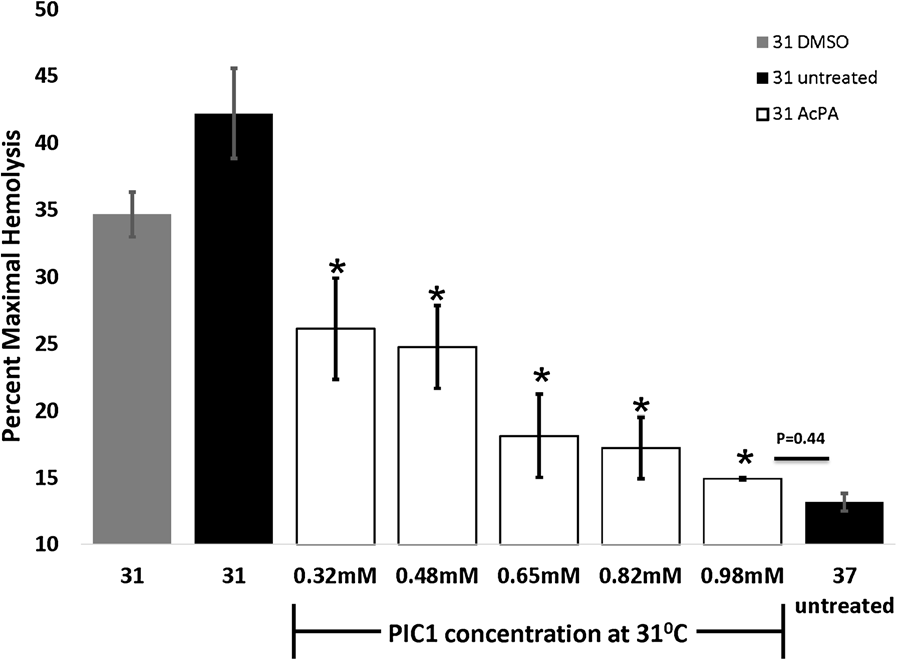
**Peptide inhibitor of C1 (PIC1) and hypothermia-enhanced complement-mediated hemolysis.** Human serum (16.5%) was pre-incubated with 0.98 mM, 0.82 mM, 0.65 mM, 0.48 mM or 0.32 mM PIC1, DMSO vehicle control, or buffer control before performing the CH50-type assay at 31° or 37°C. PIC1 inhibited hypothermia-enhanced complement activation and hemolysis at 31°C for all doses tested up to 60% inhibition for 0.98 mM PIC1 compared with 31°C untreated (*ANOVA P <0.01). Increasing concentrations of PIC1 showed a significant dose dependent inhibition of hypothermia-enhanced complement activation at 31°C for all doses tested. At 31°C, 0.98 mM of PIC1 inhibited complement mediated cell lysis to a level equivalent with euthermia (37°C) (*t*-test P = 0.44) Data are mean ± SEM for 3 independent experiments.

In order to further elucidate the role of C1 in antibody-initiated hypothermia-enhanced complement activation, we tested a specific inhibitor of C1 activation, PIC1, (Peptide Inhibitor of complement C1) [23,24,27,28]. In the CH50-type assay, PIC1 successfully inhibited hypothermia-enhanced complement activation at 31°C in a dose-dependent manner by up to 60% compared with untreated control (ANOVA P = < 0.01) (Figure 5). At 0.98 mM PIC1 inhibited hypothermia-enhanced (31°C) complement-mediated cell lysis to a level not statistically different from that for euthermia (37°C) (*t*-test P = 0.44). These results suggest that hypothermia-enhanced antibody-initiated complement activation can be blocked using a specific inhibitor of C1 activation.

## Discussion

**Figure 6:**
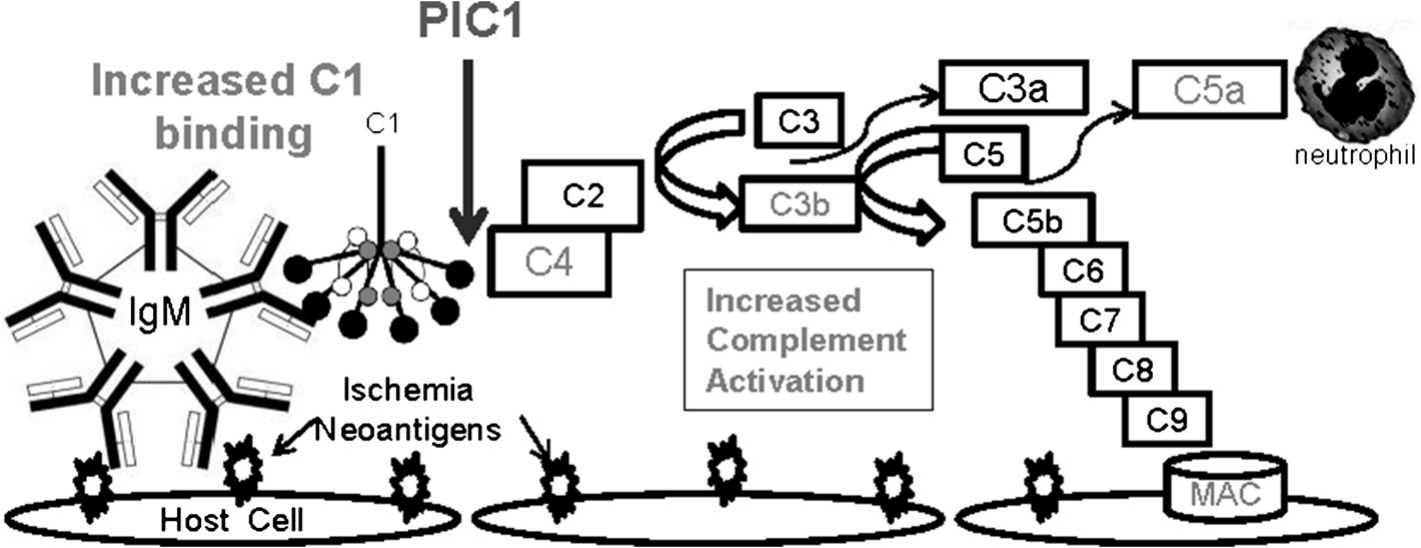
**Model of antibody-initiated complement activation in ischemia reperfusion injury and hypothermia effects on complement activation.** Hypoxic insult induces expression of 'neoantigens' on the surface of vascular endothelial cells. These neoantigens are recognized by natural antibodies (IgM) initiating complement activation leading to downstream inflammatory effectors. Therapeutic hypothermia temperatures were shown to increase C1/C1q binding, increase opsonization with C4-fragments and C3-fragments, increase C5a anaphylatoxin generation, and increase eukaryotic cell lysis via membrane attack complex (MAC) formation. Increases in complement function demonstrated in this study are shown in grey. PIC1 inhibits complement activation at C1 preventing C4 activation.

The clinical benefit of therapeutic hypothermia in moderating IRI and improving neurological outcomes is modest, but clear [31,32]. Therapeutic hypothermia offers an 11% reduction in risk of death or disability in neonatal hypoxic ischemic brain injury, a decrease from 58% to 47% [33]. Therapeutic hypothermia appears to have beneficial effects for several mechanisms that likely contribute to the pathogenesis of IRI, but all of the effects of cooling the human body are unknown. It is reasonable to expect that a non-targeted treatment such as therapeutic hypothermia may have some unrecognized detrimental actions, which could partially attenuate the positive effects. The most striking finding from our studies is that therapeutic hypothermia temperatures increase antibody-initiated complement activation enhancing inflammation, opsonization, and destruction of eukaryotic cells *in vitro*. Thus, our data strongly suggests that the therapeutic effect of hypothermia may not be mediated by reducing complement activation, but rather, clinical benefit is derived via other mechanisms. These data suggest that therapeutic hypothermia temperatures likely increase antibody-initiated complement activation *in vivo* (Figure 6), potentially partially attenuating the clinical benefits of therapeutic hypothermia in the treatment of IRI.

Our data show that increased complement activation at lower temperatures was mediated by antibody-initiated complement activation, but the alternative pathway was unaffected by the temperatures tested. Therapeutic hypothermia temperatures increased complement-mediated cell lysis demonstrating enhanced membrane attack complex pore formation. At lower temperatures there was increased C5a generation, which recruits and activates neutrophils enhancing local inflammation. Increased iC3b opsonization of eukaryotic cells occurred at lower temperatures, which targets them for attack by phagocytic cells. Increased C4 activation at low temperatures suggested increased activation at C1, which cleaves C4. Testing activation of the enzymatic components of C1 showed decreased enzymatic activity at lower temperatures, which is a common property of enzymatic reactions. However, multiple assays testing C1 or C1q binding showed a consistent trend toward increased binding to antibody-sensitized cells at lower temperatures. Increased binding of molecules often occurs at lower temperatures [34] and suggested that increased binding of C1 may be responsible for increased antibody-initiated complement activation despite decreased enzymatic activity. Increased antibody-initiated complement activation was not mediated by temperature effects on antibody-binding, because all antibody-sensitization procedures were performed at 30°C. Using serum from a patient with cutaneous vasculitis, it was found that cryoglobulin increased activation of the complement system at 20°C [35].

It is notable that antibody-initiated complement activation was decreased at 41°C compared with 37°C. We speculate that decreased antibody-initiated complement activation at febrile temperatures may be a mechanism of dampening classical pathway activation after systemic inflammation with cytokines and TNF has been achieved. Classical pathway activation occurs extremely early in inflammation and once initiated, complement activation will be perpetuated by the positive-feedback loop of the alternative pathway, which does not appear to be affected by febrile temperatures. Thus, this may be a down-regulatory mechanism to decrease further activation of the classical pathway and moderating potential complement-mediated damage to the host. Although mechanistically different, fever decreasing classical pathway activation may be functionally similar to soluble TNF-receptor generation down-regulating TNF effects late in inflammation [36,37].In order to elucidate the mechanism of hypothermia-enhanced antibody-initiated complement activation, we tested a specific inhibitor of C1 activation (Figure 6). PIC1 successfully inhibited hypothermia-enhanced complement activation at 31°C to a level similar to that which occurred at euthermia (37°C), consistent with C1-mediated activation.

Our findings differ from a study demonstrating attenuated complement activation following hypothermia *in vivo* in a cohort of cardiopulmonary bypass patients [38]. However, the findings in the study cannot be attributed to hypothermia alone, since the practices of hemodilution and heparinization in cardiopulmonary bypass utilized in this study likely confound the association as they are independent causes of complement inhibition [39]. One of the limitations of this study is that it does not model IRI. However, this limitation allowed us to explore the independent effect of hypothermia on antibody-initiated complement activation. Future directions include testing the effects of hypothermia in the Vannucci model of rat brain IRI [40] and elucidating the contribution of the lectin and classical pathway in hypothermia enhanced complement activation.

## Conclusions

Therapeutic hypothermia temperatures increased antibody-initiated complement activation and eukaryotic cell destruction suggesting that the benefits of therapeutic hypothermia may be mediated via other mechanisms. A peptide inhibitor of complement (PIC1) significantly inhibited this enhanced complement activation. Antibody-initiated complement activation has been shown to contribute to ischemia-reperfusion injury in several animal models, suggesting that for diseases with this mechanism hypothermia-enhanced complement activation may partially attenuate the benefits of therapeutic hypothermia. Future directions include testing the relationship between complement activation and hypothermia in an animal model of neonatal hypoxic-ischemic encephalopathy.

## Competing interests

The authors have no conflicts of interest or financial disclosures to report with regard to this manuscript. PIC1 and its derivatives are currently protected under US Patent 8,241,843 and Patent Pending 13/809371.

## Authors’ contributions

Conceived the ideas for this study NKK, KMC; Acquisition, analysis and interpretation of data TAS, CTM, PSH, AS, MPS, WTB, NKK, KMC; Wrote the manuscript TAS, NKK, KMC; All authors read and approved the final manuscript.
